# TIL cell therapy in HIV positive patient with metastatic melanoma: case report

**DOI:** 10.3389/fonc.2026.1818578

**Published:** 2026-05-20

**Authors:** Brian Whetsell, Juan Alban, Adam Y. Lin, Jeffrey D. Wayne, Sunandana Chandra

**Affiliations:** 1 Department of Comparative Human Development, The University of Chicago, Chicago, IL, United States; 2 McGaw Medical Center, Northwestern University, Chicago, IL, United States; 3 Division of Hematology Oncology, Department of Medicine, Feinberg School of Medicine, Northwestern University, Chicago, IL, United States; 4 Robert H. Lurie Comprehensive Cancer Center of Northwestern University, Chicago, IL, United States; 5 Department of Surgery, Northwestern University Feinberg School of Medicine, Chicago, IL, United States

**Keywords:** case report, cell therapy, HIV, immunotherapy, melanoma, TIL, metastatic melanoma

## Abstract

Tumor-infiltrating lymphocyte (TIL) therapy is an emerging treatment for patients with metastatic melanoma whose disease has progressed on immune checkpoint inhibitors. However, individuals living with well-controlled human immunodeficiency virus (HIV) have historically been excluded from clinical trials evaluating adoptive cell therapies, leaving uncertainty regarding safety, tolerability, immune effects, and oncologic response in this population. We present the first known case of a patient with virologically suppressed HIV receiving TIL therapy for immunotherapy-refractory metastatic melanoma. A 37-year-old man with long-standing HIV on antiretroviral therapy (ART) developed rapidly progressive, BRAF-V600E mutant metastatic melanoma involving bulky axillary disease and pulmonary metastases despite anti–PD-1 therapy, combined checkpoint blockade, and BRAF/MEK–targeted therapy. His course prior to TIL therapy was notable for minimal treatment-related toxicities and intermittent detectable low-level HIV viremia. After multidisciplinary review, he underwent TIL harvest followed by lymphodepleting chemotherapy, lifileucel infusion, and high-dose interleukin-2 (IL-2). TIL therapy was tolerated with expected toxicities, including grade 1 cytokine release syndrome and IL-2–associated grade 3 hypotension requiring brief vasopressor support. At day 44, imaging demonstrated a partial response with decreased pulmonary metastases and stabilization of axillary disease. Progression of disease was observed by day 86. CD4 counts fluctuated markedly throughout treatment, though virologic suppression was ultimately restored by day 100. This case demonstrates that TIL therapy can be feasibly administered to a patient with well-controlled HIV, with manageable toxicity and early radiographic response. These findings underscore the need for prospective inclusion of people living with HIV in cellular therapy trials to better characterize safety, immune dynamics, and predictors of durable benefit.

## Introduction

Melanoma remains a leading cause of cancer-related mortality, with outcomes largely dependent on stage at diagnosis and responsiveness to immune-based therapy. Immune checkpoint inhibitors (ICIs) have transformed the management of advanced melanoma, yet up to 40% of patients demonstrate primary resistance, and many others develop acquired resistance despite combination immunotherapy (1). For these individuals, treatment options have historically been limited, particularly for those who have also exhausted targeted therapy approaches.

Tumor-infiltrating lymphocyte (TIL) therapy represents a significant advancement in the management of immunotherapy-refractory melanoma. Lifileucel, the first FDA-approved TIL product, leverages ex vivo expansion of autologous lymphocytes harvested from tumor tissue, followed by reinfusion in conjunction with lymphodepleting chemotherapy and high-dose interleukin-2 (2). Clinical studies have demonstrated objective response rates exceeding 30% in heavily pre-treated patients (3). However, individuals living with human immunodeficiency virus (HIV), even those with well-controlled disease, were excluded from pivotal trials due to concerns regarding immune competence, viral reactivation, and tolerability of lymphodepleting regimens and IL-2.

Despite these exclusions, accumulating evidence suggests that patients with well-controlled HIV can safely receive ICIs and may experience outcomes comparable to HIV-negative individuals (4). Similarly, the use of CAR-T therapy in HIV patients with relapsed or refractory lymphoma has been proven to be safe and effective (5). Whether this safety profile extends to adoptive cell therapies in metastatic melanoma remains unknown. The interaction between HIV-associated immune dysregulation, CD4/CD8 dynamics, and TIL expansion or persistence has not been previously described.

We report a case of TIL therapy administered to a patient with virologically suppressed HIV and metastatic melanoma refractory to immunotherapy and targeted therapy. This report provides insight into treatment tolerability, immune and virologic parameters, and short-term oncologic outcomes, contributing needed real-world evidence in a population historically excluded from cellular therapy trials.

## Background

People living with HIV (PWH) are at increased risk of skin cancers, including cutaneous squamous cell carcinoma and melanoma. In this context, emerging population-level data also suggest worse melanoma outcomes. An analysis from the HIV/AIDS Cancer Match (HACM) study found that melanoma-specific mortality is notably higher among PWH (6). Cutaneous melanoma remains a major public health burden. It is the fifth most common cancer in the United States, with an estimated 100,000 new diagnoses projected for 2024 (7). Approximately 5% of patients present with stage IV metastatic melanoma, for whom the 5-year relative survival remains 35%. Nevertheless, outcomes for stage IV melanoma have improved significantly over the past decade, with nearly a 20% increase in survival since 2015 attributable to advances in targeted therapies and immunotherapy (8). Melanoma’s long-recognized sensitivity to lymphocyte infiltration, ultimately led to the development of immune checkpoint inhibitors (ICIs) which now form the backbone of therapy for advanced disease (9, 10).

In the unresectable or metastatic setting, standard first-line treatment includes combination immunotherapy with nivolumab (anti–PD-1) plus ipilimumab (anti–CTLA-4) or nivolumab plus relatlimab (anti–LAG-3). Monotherapy with nivolumab or pembrolizumab (anti-PD-1) or for individuals with BRAF V600 mutations, dual BRAF/MEK inhibition are alternative options in select cases, though responses are often limited in duration (9, 11). For patients with metastatic melanoma refractory to ICI’s, tumor-infiltrating lymphocyte (TIL) therapy has emerged as a novel later-line strategy for patients with metastatic melanoma refractory to ICIs. Lifileucel consists of autologous T cells harvested from a patient’s tumor, expanded ex vivo, infused following lymphodepleting chemotherapy, and subsequently supported by high-dose IL-2 to promote *in vivo* activation and persistence (3, 12). Despite its promise, the intense immunologic manipulation inherent to TIL therapy raises concerns for individuals with preexisting immune compromise.

Patients living with well-controlled, virologically suppressed human immunodeficiency virus (HIV) have historically been excluded from oncology clinical trials, including the pivotal TIL trial that led to FDA approval (3). Nevertheless, emerging evidence demonstrates that individuals with undetectable HIV can safely receive ICIs and may respond comparably to HIV-negative patients (13). Similarly, the use of CAR-T therapy in HIV patients with relapsed or refractory lymphoma has been reported to be safe and effective (5). Mechanistically, HIV infection drives chronic immune activation and durable quantitative and qualitative remodeling of the T-cell compartment, including upregulation of immune checkpoint receptors (ICRs) such as PD-1, TIM-3, and TIGIT on both CD4+ and CD8+ T cells with associated functional impairment. Although antiretroviral therapy (ART) reduces ICR expression, emerging data suggest only partial reversal of HIV-associated epigenetic and phenotypic reprogramming. Notably, expansion of the terminally differentiated effector memory (T_EMRA_) subset can persist even after three years of ART, consistent with durable reshaping of the T-cell pool and/or ongoing low-level antigenic stimulation that promotes T_EMRA_ differentiation (14). How these immune alterations affect tolerability and efficacy of tumor-infiltrating lymphocyte (TIL) therapy remains unknown, as no published data describe its use in this population.

This report presents the first known case of a patient with well-controlled HIV undergoing TIL therapy for metastatic melanoma. We describe the clinical presentation, therapeutic decision-making, and 100-day post-infusion outcomes in a 35-year-old man on antiretroviral therapy (ART). By documenting this experience, we aim to contribute to the limited understanding of cellular immunotherapy in individuals living with HIV and to inform future clinical and research considerations.

## Case presentation prior to consideration of TIL

Our patient is a 37-year-old Caucasian man with a past medical history significant for a prior wide local excision of melanoma of unknown stage on his right lower back in early 2000, and human immunodeficiency virus (HIV) diagnosed in 2010. At the time of his HIV diagnosis, his CD4 count was greater than 500.

In early 2010, he initiated antiretroviral therapy (ART) with atazanavir, boosted with ritonavir, in combination with emtricitabine/tenofovir. Before initiating therapy, his CD4 count was 314 cells/mm³ and HIV viral load was 10,397 copies/mL. By mid-2010, his CD4 count was 380 with an undetectable viral load. At the time of his presentation in mid-2023, he was taking bictegravir/emtricitabine/tenofovir alafenamide (Biktarvy).

**Figure 1:**
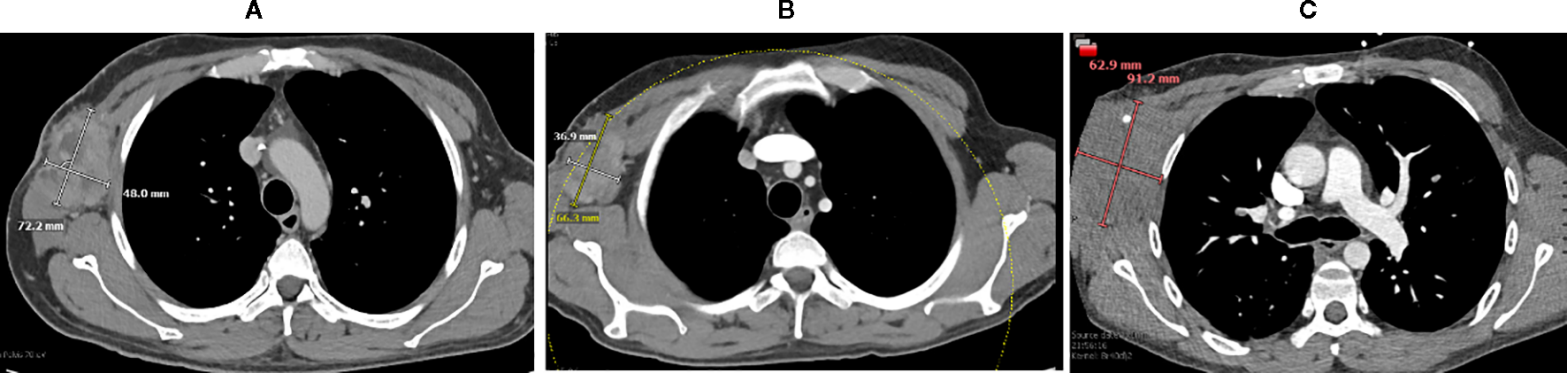
Right axillary lesion and radiographic responses to TIL. **(A)** CT chest obtained prior to TIL infusion (September 2024) demonstrating the right axillary mass measuring 72.2 mm × 48 mm. **(B)** CT chest obtained two months after TIL therapy (December 2024) demonstrating interval decrease in lesion size to 66.3 mm × 36.9 mm. **(C)** CT chest obtained five months after TIL therapy (February 2025) demonstrating interval enlargement of the lesion to 91.2 mm × 62.9 mm. These images correspond to the representative axillary mass shown in Image 1.

In January 2023, the patient presented with several days of self-detected right axillary lymphadenopathy. Ultrasound revealed two abnormal lymph nodes concerning for malignancy, and biopsy confirmed melanoma with a BRAF V600E mutation. Staging PET/CT and brain MRI demonstrated two hypermetabolic right axillary nodes (largest 4.7 × 3.3 cm) without distant metastatic disease. Given the size and vascularity of the masses, he began neoadjuvant pembrolizumab (per SWOG 1801); however, after three cycles, the dominant node rapidly enlarged to 8.2 × 7.5 cm. Therapy was escalated to ipilimumab plus nivolumab, but imaging after three cycles again showed rapid tumor growth to 12.3 × 12.1 cm, accompanied by worsening right upper-extremity neuropathic pain. He received a single fraction of palliative radiation (15 Gy), though follow-up CT four weeks later demonstrated further enlargement to 15.8 × 12.1 cm, and additional radiation was not recommended. The right axillary mass, which served as a representative lesion and the site of TIL harvest, is tracked in **Figure 1**.

**Figure 2:**
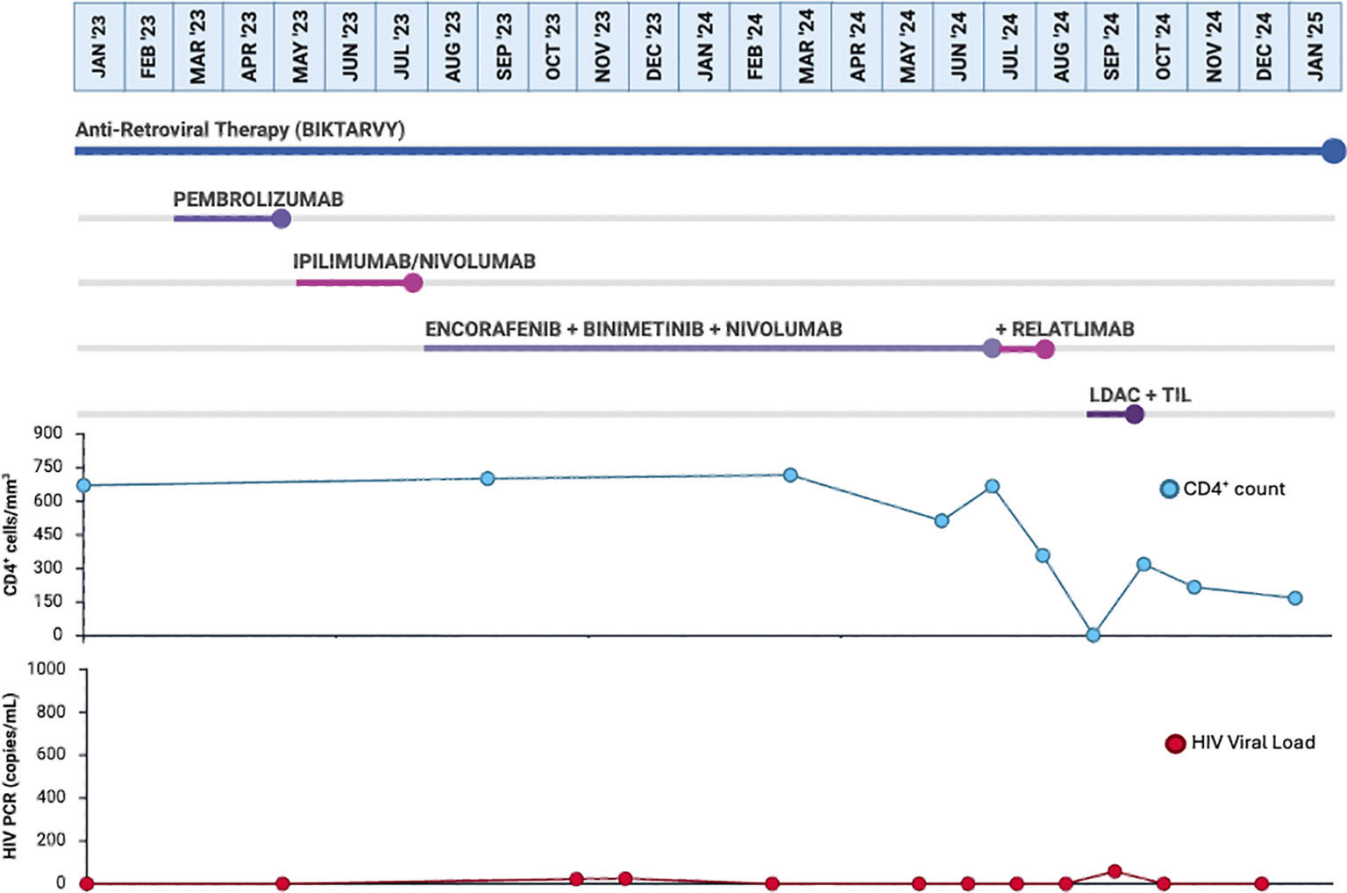
Timeline of therapies prior to TIL. This image depicts the clinical timeline for the patient from the diagnosis of metastatic melanoma through the end of the 100-day post–TIL therapy follow-up period. The timeline shows the sequence and duration of all systemic anticancer treatments, including pembrolizumab, ipilimumab/nivolumab, BRAF/MEK–targeted therapy, nivolumab/relatlimab, lymphodepleting chemotherapy, TIL infusion, and high-dose interleukin-2. Corresponding CD4+ T-cell counts and HIV viral load are displayed at matched time points to illustrate immune trends during systemic therapy, lymphodepletion, and post-TIL immune reconstitution. The image provides an integrated overview of treatment exposure, HIV viral load and CD4+ dynamics throughout the patient’s clinical course.

Given continued progression, he was transitioned to triplet therapy with encorafenib/binimetinib plus nivolumab. This course was complicated by visual disturbances (halos, floaters), pneumonia with sepsis and acute hypoxemic respiratory failure, and acute kidney injury related to hypovolemia from diarrhea. After 15 weeks, his HIV viral load became detectable (21.9 copies/mL), likely due to drug–drug interactions between encorafenib and his ART regimen; cancer therapy and ART were continued without changes, and viral levels were monitored closely. After 30 weeks of triplet therapy, imaging demonstrated a response with regression of the axillary mass to 3.2 × 2.7 cm. Unfortunately, by week 45—after 11 cycles of nivolumab—repeat imaging again showed enlargement of the mass to 5.7 × 3.8 cm and new bilateral pulmonary nodules. At week 47, his HIV viral load again became undetectable and his CD4 count was 513 cells/mm³. In light of progressive disease despite multiple lines of therapy, he was evaluated for tumor-infiltrating lymphocyte (TIL) therapy, and quad therapy with encorafenib/binimetinib plus nivolumab/relatlimab was initiated; however, nivolumab/relatlimab was discontinued after a single cycle due to insurance denial. A detailed overview of the patient’s systemic therapies, CD4 T-cell trends, HIV viral load, and timing of TIL harvest and infusion is shown in the clinical timeline (**Figure 2**).

## Case presentation during TIL

Approximately ten days after TIL harvest, his HIV viral load became detectable at <20 copies/mL. Encorafenib/binimetinib was discontinued four weeks before TIL infusion as per protocol. Two weeks before lymphodepleting chemotherapy, he developed an acute, diffuse rash and oropharyngeal thrush, which resolved within 10 days after treatment with fluconazole, clotrimazole lozenges, antihistamines, and topical tacrolimus.

The patient was admitted in late 2024 for TIL infusion. On day –7, his CD4 count was 358 cells/mm³. He received lymphodepleting chemotherapy consisting of cyclophosphamide and mesna on days –7 and –6, followed by fludarabine on days –5 through –1. A CT scan obtained on day –5 revealed an axillary mass measuring 7.2 × 4.8 cm, an enlarged right hilar lymph node (1.7 cm), and progression of multiple pulmonary nodules (largest 2.3 cm). Expected toxicities of lymphodepletion occurred, including elevated liver transaminases, hypoalbuminemia, hypophosphatemia, febrile neutropenia, leukopenia, and anemia.

On day 0, with a CD4 count of 3 cells/mm³, he received Lifileucel infusion. Infusion-related symptoms included headache, diffuse myalgias, tachycardia, and fever to 101.1°F, consistent with grade 1 cytokine release syndrome. Cultures were negative, though he received empiric piperacillin/tazobactam for six days for possible neutropenic fever.

High-dose interleukin-2 (IL-2) began on day +1. After the first dose, he developed borderline hypotension, tachycardia, and fever, requiring IV fluids and midodrine. After the second dose, administered early on day +2, he developed worsening hypotension (nadir 70/40 mmHg). Due to inadequate response to fluids, midodrine, and dexamethasone, he required norepinephrine and was transferred to the ICU. Upon ICU transfer, the patient was started on stress dose steroids. Pressors were weaned and hemodynamics stabilized by day +3; IL-2 was resumed that evening. He received four additional doses of IL-2, two on day +3 and two on day +4. Stress dose steroids were discontinued on day +4. Per protocol, the sixth IL-2 dose was withheld due to grade 3 hypotension requiring ICU-level care; however, this event was likely multifactorial given the patient’s pre-existing adrenal insufficiency. The remainder of his admission (days +5 to +7) was unremarkable, and he was discharged in stable condition. Of note, his HIV medication was continued during the entirety of this admission.

Post-discharge, his CD4 count and HIV viral load fluctuated as anticipated following lymphodepletion and IL-2. On day +15, his CD4 count was 468 cells/mm³ with an HIV viral load of 57.7 copies/mL. By day +22, his viral load again became undetectable.

Day +44 restaging CT demonstrated partial response, with decreased axillary mass size.

(6.6 × 3.7 × 8.3 cm), decreased hilar lymphadenopathy (0.8 cm), and interval decrease in pulmonary nodules (largest 1.2 cm). By day +51, his CD4 count had decreased to 217 cells/mm³, with a viral load <20 copies/mL.

By day +86, systemic imaging demonstrated disease progression, with the axillary mass measuring 8.1 × 2.9 × 6.9 cm, an increase in hilar lymphadenopathy to 1.4 cm, and enlargement of two pulmonary nodules (largest 2.2 cm). Representative CT chest images before TIL therapy, two months after infusion, and five months after infusion demonstrate the initial radiographic improvement and subsequent progression of this right axillary lesion (**Figure 1**). At day +100, his CD4 count was 168 cells/mm³, and his HIV viral load was again undetectable.

Following post-TIL disease progression, the patient underwent multiple additional lines of therapy. However, none resulted in prolonged disease control. He received quadruplet therapy with nivolumab, relatlimab, encorafenib, and binimetinib for two months as a bridge to a subsequent clinical trial combining anti–IL-8, anti–PD-1, and stereotactic body radiation therapy (SBRT), but he experienced continued progression. He was subsequently treated with standard-of-care carboplatin and paclitaxel, followed by re-challenge with encorafenib and binimetinib. Despite these interventions, his disease continued to progress, and he died on day +358 after approximately 3 weeks on the final regimen.

## Conclusions/discussion

Tumor-infiltrating lymphocyte (TIL) therapy has emerged as an important treatment option for patients with unresectable or metastatic melanoma whose disease has progressed on immune checkpoint inhibitors (ICIs) and, when applicable, targeted therapy. Clinical studies have demonstrated an objective response rate (ORR) of 31.4% with Lifileucel in heavily pretreated melanoma (3). However, these findings do not include individuals living with HIV, who were excluded from the pivotal trials that led to approval of TIL therapy. As a result, it remains unknown whether well-controlled HIV infection influences the safety, efficacy, or durability of responses to TIL therapy.

Preclinical and translational data suggest that favorable melanoma outcomes correlate with the presence and functionality of intratumoral T cells, as well as the degree of TIL infiltration within tumor tissue (12). Because HIV infection directly affects CD4+ T-cell populations and modulates CD8+ T-cell dynamics, a theoretical concern exists that TIL expansion, persistence, or antitumor activity may differ in individuals with HIV compared with the general population. However, evidence supporting such differences remains limited. For example, in a study evaluating tumor-infiltrating immune cells in Chinese patients with HIV and colorectal cancer, no significant differences in the densities of intratumoral CD4+ or CD8+ immune infiltrates were observed compared with HIV-negative controls (15). Whether these findings translate to melanoma, a malignancy known to be more common among individuals with HIV is unclear, given the distinct immunologic mechanisms that underlie melanoma tumorigenesis relative to colorectal cancer (16).

In this case, the patient’s CD4 count, and HIV viral load fluctuated throughout the pre- and post-TIL periods. While transient detectable viremia predated TIL therapy and was likely influenced by drug–drug interactions with encorafenib, the marked cytopenias and CD4 decline after lymphodepleting chemotherapy and IL-2 are expected consequences of TIL preparative regimens rather than HIV-specific pathology. Importantly, his HIV viral load returned to undetectable levels by day +22 and remained suppressed through day +100, suggesting that TIL therapy and high-dose IL-2 did not precipitate sustained viral rebound. Nevertheless, longer-term follow-up would be needed to determine whether immune reconstitution in this setting ultimately resembles that of HIV-negative TIL recipients. Additionally, further studies would be needed to determine if TIL therapy led to any additional changes in the quantitative and qualitative remodeling of the T-cell population as described earlier.

From an oncologic perspective, our patient’s treatment course prior to TIL underscores the heterogeneity of melanoma responses to modern therapy. Up to 40% of patients exhibit primary resistance to anti–PD-1 therapy (17), but progression despite subsequent combination ipilimumab and nivolumab is less common (18). His mixed response to triplet targeted therapy with encorafenib/binimetinib plus nivolumab, followed by renewed progression at 45 weeks, aligns with data showing that BRAF/MEK inhibition often produces responses of limited duration (11, 19). Given his ineligibility for clinical trials due to HIV status, TIL therapy represented the only remaining standard-of-care option.

The patient tolerated TIL therapy with expected toxicities, including grade 1 cytokine release syndrome and IL-2–associated hypotension requiring ICU admission. Similar hemodynamic events occur in HIV-negative patients receiving TIL therapy; thus, his toxicity profile does not suggest increased susceptibility attributable to HIV status. In this case, earlier initiation of stress-dose steroids may have mitigated the severity of hypotension and potentially avoided ICU transfer given the patient’s history of adrenal insufficiency. However, the patient was hesitant to escalate steroid dosing because of prior steroid-related toxicities. On day +44, he demonstrated an early radiographic response with decreased pulmonary nodules and stable-to-improved axillary disease, consistent with the response kinetics observed in clinical trials. However, by day +86, imaging showed disease progression. The phenomenon of early response followed by relapse has been described among TIL recipients, highlighting the need for ongoing research into mechanisms of TIL persistence, exhaustion, and tumor immune evasion (20).

Although this case describes only a single individual, it provides important preliminary insight into the feasibility of administering TIL therapy to patients with well-controlled HIV. The safety profile appears consistent with that observed in the general melanoma population. Short-term virologic stability was preserved, and the patient experienced an initial objective response to therapy. Whether HIV-associated immune dysregulation may influence the durability of TIL responses warrants further investigation.

TIL therapy is an important addition to the treatment landscape for advanced melanoma, particularly for patients who have exhausted standard immunotherapy and targeted therapy options. Although immunotherapy trials have increasingly included patients with well-controlled HIV, the expanding role of cellular therapies in melanoma highlights the need for intentional inclusion of people living with HIV (PWH) in prospective trials (21). Encouragingly, meaningful progress has been made in recent years: Reuss et al. demonstrated that Cancer Therapy Evaluation Program (CTEP) advocacy increased inclusion of PWH in anti–PD-1/PD-L1 trial protocols from 16% at the letter-of-intent stage to 70% in final approved protocols (21). Such inclusion is important not only for improving the generalizability of clinical trial results, but also for generating the evidence needed to support insurance coverage and equitable access to emerging therapies for PWH.

## Data Availability

The original contributions presented in the study are included in the article/supplementary material. Further inquiries can be directed to the corresponding author.
